# Regional disparities in antenatal care utilization among pregnant women and its determinants in Ethiopia

**DOI:** 10.3389/fgwh.2024.1230975

**Published:** 2024-02-09

**Authors:** Nefsu Awoke, Sabit Abazinab Ababulgu, Lolemo Kelbiso Hanfore, Eyasu Gambura Gebeyehu, Senahara Korsa Wake

**Affiliations:** ^1^ School of Nursing, College of Health Science and Medicine, Wolaita Sodo University, Wolaita Sodo, Ethiopia; ^2^ Jimma Town Health Office, Jimma, Ethiopia; ^3^ School of Nursing, College of Medicine and Health Science, Wachemo University, Hossana, Ethiopia; ^4^ School of Public Health, College of Health Science and Medicine, Wolaita Sodo University, Wolaita Sodo, Ethiopia; ^5^ Department of Statistics, College of Natural and Computational Science, Ambo University, Ambo, Ethiopia

**Keywords:** antenatal care, number of visits, poisson regression, DHS, Ethiopia

## Abstract

**Background:**

Antenatal care primarily focuses on health care checkups, the provision of advice on healthy behaviors, and the delivery of psychological, social, and emotional support for women with pregnancy. The national target set by the Ethiopian government is to achieve 95% of at least four ANC visits. Nevertheless, 43.11% of women had four or more checkups, according to the 2019 Ethiopian Demographic and Health Survey. Despite this achievement, antenatal care visits differ significantly between Ethiopian regions. Consequently, the purpose of this study was to assess regional disparities in pregnant women's utilization of antenatal care and its determinants in Ethiopia.

**Methods:**

We have used 2019 intermediate Ethiopian Demographic and Health Survey data for analysis. The analysis comprised a total of 3,917 weighted women age 15–49 who had a live birth in the 5 years preceding the survey. Poisson regression analysis was done using SAS software version 9.4. To show the strength and direction of the association, an incidence rate ratio with a 95% confidence interval was used. Variables with a *p*-value <0.05 were declared as significant factors associated with the number of ANC visits.

**Results:**

In Ethiopia, the number of ANC visits differs between regions. With a mean of 4.74 (95% CI: 4.49, 4.99), Addis Ababa reported the highest percentage of ANC visits (82.7%), while the Somali Region reported the lowest percentage (11.3%) with a mean of 0.73 (95% CI: 0.57, 0.88). Maternal age, educational level, religion, household wealth index, place of delivery, and household size show significant associations with the number of antenatal care visits.

**Conclusions:**

In Ethiopia, there is the highest regional disparity in the number of ANC visits. The number of ANC visits was influenced by the mother's age, education, religion, household wealth index, place of delivery, and household size. Regarding the ANC visits, there should be initiatives that address the demands of pastoralist and agro-pastoralist communities to increase ANC utilization. As with many other health outcomes, education and low socio-economic status were associated with low ANC visit but these are tied to the overall social development of a country and are not immediately amenable to public health interventions

## Introduction

### Background

Antenatal care (ANC) utilization is defined as the receipt of care by pregnant women from skilled health care provider initiated within 12 weeks of gestation and frequent visits (at least four ANC visits) for care related to pregnancy (1). ANC is one of the best opportunities for pregnant mothers to prevent maternal and child mortality and morbidity by increasing women's access to basic obstetric care (2). It's considered a starting point for prenatal, postpartum, and pediatric vaccination services by creating a connection between the clinic and the healthcare practitioner for future actions (3, 4). ANC majorly focuses on health care checkups, the provision of advice on healthy behaviors, and the delivery of psychological, social, and emotional support for women with pregnancy (5). Also, it has paramount importance in reducing complications related to pregnancy (6). Moreover, antenatal care offers services such as tetanus immunization, hypertension management to prevent eclampsia, vitamin supplements, and other services (7). The care is primarily delivered by trained and skilled health care professionals, such as doctors, nurses, midwives, and other accredited health care professionals (5).

Global maternal deaths decreased from 451,000 to 295,000 between 2000 and 2017 (8). Even though maternal mortality is showing progress in reduction, it still becomes a major health problem in some parts of the world (5), with a majority (94%) occurring in developing countries and Sub-Saharan Africa sharing approximately two-thirds of maternal deaths (9, 10). In Ethiopia, the MMR was predicted to be 401 deaths per 100,000 live births in 2017, according to UN estimations (10).

The global community has given due emphasis to reducing maternal deaths. For instance, MDG Goal 5 focused on reducing the death rate by 75% at the end of 2015, but the overall achievement was only 44%. Lowering maternal mortality was one of the Sustainable Development Goals (SDGs), which were adopted in the wake of the Millennium Development Goals. By 2030, SDG3.1 targets global maternal death rates of less than 70 per 100,000 live births by the year 2030 (11). Also, in an effort to reduce maternal mortality, WHO revised the recommended ANC visit from 4 to 8 or more contacts, with the first ANC contact in the first 12 weeks of gestation and subsequent contacts at 20, 26, 30, 34, 38, and 40 weeks of gestation to improve the effectiveness, timing, and quality of care (11, 12).

Pregnant women without complications are advised by the World Health Organization (WHO) to have four ANC checkups throughout their pregnancy (10). Only three out of five women attended at least four antenatal care visits, according to the global 2017 survey. Only 52% of women in sub-Saharan Africa, where maternal mortality is high, had at least four ANC visits (2). In Ethiopia, 43.11 percent of women had four or more checkups, according to the 2019 Ethiopian Demographic and Health Survey (EDHS) (13), which is much lower than the target set by the Ethiopian Government to achieve 95% of at least four ANC visits (12).

Well-timed ANC utilization reduces direct and indirect causes of maternal death, but there are different factors that determine the utilization, such as maternal age (3, 14), women's education (3, 8, 13, 14), pregnancy status (3), the husband's occupation (3, 13, 14), socioeconomic status (13), the wealth index (3, 8, 14), accessing radio (3, 14), the number of children (3, 8, 15), the place of residence (3, 8, 13, 14), understaffed health facilities (13), and distant to health facilities (8, 13–15).

According to the 2019 Ethiopian DHS report, just 43% of pregnant women had the recommended four visits, and 48% had given birth to their most recent children in health facilities, even though 74% of pregnant women had at least one ANC visit with a qualified provider (10). Despite the observed benefits of appropriate ANC utilization, its use remains low in Ethiopia, with significant variations across regions. Consequently, the purpose of this study was to assess regional disparities in pregnant women's utilization of antenatal care and its determinants in Ethiopia.

## Methods

### Data source

The Demographic and Health Survey (DHS), which was carried out in Ethiopia, served as the study's data source. Along with the Central Statistics Agency, the Ethiopian Public Health Institute (EPHI) carried out the study (16). From March 21, 2019, to June 28, 2019, estimates at the national, regional, and local levels, as well as for urban and rural areas, were used to conduct the survey on women of reproductive age (ages 15–49) (13). Data were accessed from their URL: www.dhsprogram.com, with a reasonable request. Specifically, women's files (IR) were extracted and used for this study.

### Sampling procedure

The sample for the 2019 Ethiopia Mini-DHS was stratified and chosen in two stages. After dividing Ethiopia's nine regions and two administrative cities into urban and rural areas, there are 21 sampling strata. In two stages, samples of enumeration areas (EAs) were chosen individually in each stratum. 200 EAs from eight regions and 105 EAs from three regions (Oromia, SNNPR, and Amhara) were chosen for the first stage, for a total of 305 EAs (93 in urban areas and 212 in rural areas) based on the probability proportional to EA size and with independent selection in each sampling stratum. For the second stage's sampling frame, a list of every household in the chosen EAs was used. The second stage involved the proportionate selection of households from each EA using systematic sampling. The study's source population consisted of women of reproductive age who had given birth in the five years preceding the survey. Eligible interviewees were women of childbearing age, whether they were permanent residents or visitors who had stayed in the household the previous night. In households where multiple eligible women resided, only one woman was selected through a random lottery method to ensure the selection process was random. The weighted frequency was used for the analysis by calculated using Women's individual sample weight (6 decimals).

### Study variables

The number of antenatal visits made during the pregnancy served as the study's outcome variable (count data). Women's age, religions, present marital status, location, level of education, wealth index of the household, region, and number of children are considered as independent variables.

### Data processing and analysis

To ensure consistency with the EMDHS 2019 descriptive report, and data cleaning was done. SAS statistical software version 9.4 was used for recoding, variable formation, labeling, and data analysis. The dependent variable, ANC utilization, is count data, which is a non-negative integer. Therefore, we used the Poisson regression model for analysis. The Poisson distribution assumes that its “mean” and “variance” are equal. But this study violated this assumption since the mean and variance were 2.89 and 5.33, respectively, which suggest the presence of over dispersion. We adjusted the over dispersion and excess zeros in the data by using scale Pearson chi-square.

### Ethics statement

Ethics approval was not required as the data were freely available to the public.

## Results

### Characteristics of the respondents

In this study, 3,962 (weighted 3,917) women who had given birth in the five years preceding the survey were included. The majority, (76.3%) of the respondents were from rural areas. The mean (±SD) age of women was 28.7 ± 6.7. About, 51.3% of women did not attend formal education. Forty-two percent of women were reported to have a poor wealth index. Regarding the place of delivery 49.3% of the total births occurred in public health institutions. The mean (±SD) number of children ever born to women was 3.7 ± 2.5 (Table 1).

**Table 1:** Socio-demographic characteristics of women age 15–49 who had a live birth in the 5 years preceding the survey in Ethiopia 2019.

Variables	Category	Weighted frequency^a^	Weighted percentage
Age in 5-year age groups (in years)	15–19	227	5.8
	20–24	767	19.6
	25–29	1,190	30.4
	30–34	796	20.3
	35–39	589	15.0
	40–44	257	6.6
	45–49	89.	2.3
Place of residence	Urban	1,020	26.0
	Rural	2,897	74.0
Education Level	No education	2,010	51.3
	Primary	1,411	36.0
	Secondary	343	8.8
	Higher	153	3.9
Religion	Orthodox	1,108	28.3
	Catholic	34	0.9
	Protestant	1,153	29.4
	Muslim	1,568	40.0
	Traditional	44	1.1
	Other^b^	10	0.3
Wealth index	Poor	1,645	42
	Middle	761	19.4
	Rich	1,510	38.6
Household Size	<5	1,299	33.2
	5–10	2,381	60.8
	>=10	237	6.1
Place of Delivery	Home	1,862	47.5
	Public Health Institution	1,931	49.3
	Private Health Institution	51	1.3
	NGO health facilities	72	1.8
Parity (number of children ever born)	1–2	1,560	39.8
	3–4	998	25.5
	5 and above	1,359	34.7

^a^ A weighted frequency represents a frequency with an applied weight, essentially reflecting an estimated count in the population attributed to specific value combination.

^b^ Catholic and traditional religion follower.

### Regional disparity in ANC utilization

Utilization of antenatal care services varies among Ethiopian regions. With a mean of 4.74 ANC visits, Addis Ababa reported the highest ANC utilization, while the Somali Region recorded the lowest, with a mean of 0.73 ANC visits. Similar to this, Addis Ababa (82.7%) and Somali (11.3%) had the greatest and lowest respective percentages of pregnant women who had four or more ANC visits. Somalia had the largest percentage of women who had no ANC visits (70.8%), and Addis Ababa had the lowest percentage (3.2%) (Table 2).

**Table 2:** Regional disparity in the number of antenatal care visit among women age 15–49 who had a live birth in the 5 years preceding the survey in Ethiopia 2019.

Region	Weighted frequency	Weighted percentage	Number of ANC visit^a^				
			No visit	1–3 visit	≥4 Visit	Total	Mean (95% CI)
Tigray	285	7.3	15.1 (5.3)	87.0 (30.5)	183.0 (64.2)	285.2	3.94 (3.72, 4.15)
Afar	51	1.3	19.0 (37.2)	16.1 (31.6)	15.9 (31.2)	51.1	2.07 (1.85, 2.29)
Amhara	838	21.4	126.4 (15.1)	284.9 (34.0)	426.2 (50.9)	837.5	3.20 (3.00, 3.39)
Oromia	1,519	38.8	443.1 (29.2)	458.9 (30.2)	617.1 (40.6)	1,519.07	2.65 (2.47, 2.84)
Somali	214	5.5	151.7 (70.8)	38.5 (18.0)	24.1 (11.3)	214.4	0.73 (0.57, 0.88)
Benishangul Gumuz	47	1.2	7.9 (16.8)	12.7 (27.1)	26.3 (56.2)	46.8	3.18 (2.97, 3.38)
SNNP	786	20.1	228.3 (29.0)	289.2 (36.8)	268.8 (34.2)	786.3	2.43 (2.25, 2.61)
Gambela	19	0.5	2.6 (13.7)	10.4 (54.5)	6.1 (31.8)	19.1	2.56 (2.39, 2.73)
Harari	11	0.3	2.2 (19.4)	4.6 (41.4)	4.3 (39.2)	11.1	3.43 (3.14, 3.73)
Addis Ababa	125	3.2	3.9 (3.2)	17.7 (14.2)	103.5 (82.7)	125.19	4.74 (4.49, 4.99)
Dire Dawa	21	0.5	3.3 (15.7)	4.7 (22.2)	13.0(62.1)	21.0	4.06 (3.71, 4.41)

^a^ Includes only the most recent birth in the 5 years preceding the survey.

### Determinant of antenatal care visit among pregnant women in Ethiopia

Maternal age, educational level, religion, household wealth index, place of delivery, and household size show significant associations with the number of antenatal care visits whereas place of residence and parity were not significantly associated with the number of antenatal care visits.

The number of ANC visits was 1.23 (IRR = 1.23, 95% CI: 1.11, 1.38), 1.33 (IRR = 1.33, 95% CI: 1.18, 1.50), 1.30 (IRR = 1.30, 95% CI: 1.15, 1.48), and 1.27 (IRR = 1.27, 95% CI: 1.09, 1.48), times higher among 25–29 years, 30–34 years, 35–39 years, and 40–44 years old women than among 15–19 years old women, respectively. Compared to pregnant women with no educational level, women who attended primary school had 1.19 (IRR = 1.19, 95% CI: 1.12, 1.26), secondary school had 1.29 (IRR = 1.29, 95% CI: 1.19, 1.40), and higher education had 1.28 (IRR = 1.28, 95% CI: 1.16, 1.40) times more ANC visits. The number of ANC visits was 16% (IRR = 0.84, 95% CI: 0.79, 0.89), and 20% (IRR = 0.80, 95% CI: 0.76, 0.84) 61% (IRR = 0.39, 95% CI: 0.26, 0.82) and 50% (IRR = 0.50, 95% CI: 0.31, 0.82) lower among Protestants, Muslims, Traditionalists, and Others compared to Orthodox religion followers, respectively. There is a high rate of ANC visits among women with a middle (1.20 times higher; IRR = 1.20; 95% CI: 1.11, 1.28); or rich household wealth index (1.22 times higher; (IRR = 1.22, 95% CI: 1.14, 1.30)) compared to women with a poor house hold index. Women who live in households with more than 10 members had a 16% (IRR = 0.84, 95% CI: 0.74, 0.94) lower number of ANC visits compared to households with fewer than 5 members. Women who delivered in a public health institution had an IRR of 1.94 (95% CI: 1.83, 2.06), a private health institution had an IRR of 2.27 (95% CI: 2.02, 2.56), and NGO health facilities had an IRR of 1.45 (95% CI: 1.25, 1.68) more ANC visits than those who gave birth at home. Women from urban area had an IRR of 1.02 (95% CI: 0.99, 1.11), times higher number of visit than women from rural area. As parity increase the number of ANC visit decreases. The number of ANC visits was 3% (IRR = (0.97, 95% CI: 0.90, 1.03) and 7% (IRR = (0.93, 95% CI: 0.85, 1.01) lower among women with 3–4, and 5 and above compared to women with parity 1–2 respectively (Table 3).

**Table 3:** Multivariable poisson regression result for factors associated with the number of antenatal care visit among women age 15–49 who had a live birth in the 5 years preceding the survey in Ethiopia 2019.

Variables	Category	IRR	*p* Value
Age in 5-year age groups (in years)	15–19	1	
	20–24	1.11 (0.99, 1.24)	0.0723
	25–29	1.23 (1.11, 1.38)*	0.0002
	30–34	1.33 (1.18, 1.50)**	<0.0001
	35–39	1.30 (1.15, 1.48)**	<.0001
	40–44	1.27 (1.09, 1.48)*	0.0021
	45–49	1.12 (0.90, 1.39)	0.3260
Place of residence	Rural	1	
	Urban	1.05 (0.99, 1.11)	0.1377
Education Level	No education	1	
	Primary	1.19 (1.12, 1.26)**	<0.0001
	Secondary	1.29 (1.19, 1.40)**	<0.0001
	Higher	1.28 (1.16, 1.40)**	<0.0001
Religion	Orthodox	1	
	Catholic	0.75 (0.54, 1.04)	0.0884
	Protestant	0.84 (0.79, 0.89)**	<0.0001
	Muslim	0.80 (0.76, 0.84)**	<0.0001
	Traditional	0.39 (0.26, 0.58)**	<0.0001
	Other	0.50 (0.31, 0.82)*	0.0064
Wealth index	Poor	1	
	Middle	1.20 (1.11, 1.28)**	<0.0001
	Rich	1.22 (1.14, 1.30)**	<0.0001
Household Size	<5	1	
	5–10	0.98 (0.93, 1.04)	0.5795
	>=10	0.84 (0.74, 0.94)*	0.0025
Place of Delivery	Home	1	
	Public Health Institution	1.94 (1.83, 2.06)**	<0.0001
	Private Health Institution	2.27 (2.02, 2.56)**	<0.0001
	NGO health facilities	1.45 (1.25 1.68)**	<0.0001
Parity (number of children ever born)	1–2	1	
	3–4	0.97 (0.90 1.03)	0.3130
	5 and above	0.93 (0.85, 1.01)	0.0984

*p-value <0.05.

**p-value <0.001.

## Discussion

The result of this study indicates there is a highest regional disparity in the number of ANC visits. The highest ANC visit was reported in Addis Ababa with mean numbers of 4.74 (95% CI: 4.49, 4.99), while the lowest was reported in the Somali Region with mean numbers of 0.73 (95% CI: 0.57, 0.88). The number of ANC visits was influenced by the mother's age, education, religion, household wealth index, place of delivery, and household size.

In descending order of frequency, Addis Ababa, Tigray, Dire Dawa, Benishangul Gumuz, Amhara, Oromia, Harari, SNNPR, Gambela, Afar, and Somalia have the highest regional disparity in the number of ANC visits. In Ethiopia, more women had four or more ANC visits in 2019 compared to the previous three DHS (16, 17).

The number of ANC visits is lowest in pastoral regions in Ethiopia. Pastoralists and agro-pastoralists are found in low-land areas of Somalia, Afar, Oromia, and SNNPR (18). Since pastoralists and agro-pastoralists spend the majority of their time searching for water and grazing for their animals during the changing seasons, the static health care service strategy of Ethiopia's health care system does not match their health care demands (19). They have problems accessing health care services due to low population density, spatial mobility, dispersion, distance from health care services, and cultural factors (20). The low number of ANC visits in the Somali region is attributed to a number of factors, including a lack of funding for transportation and accommodation, a lack of awareness about ANC, a lack of husband support, and the absence of full ANC service packages because most health facilities lack the necessary staff and supplies (21, 22).

The national target of four or more ANC visits set for 2025 by the Health Sector Transformation Plan II (HSTP II) is 81% (23). The program is left with only two years but among the regions of Ethiopia according to EDHS 2019 data only Addis Ababa achieved the target while other regions have made less progress compared to Addis Ababa.

In this study, there was a strong association between education level and the number of ANC visits. Women who attended primary, secondary, or higher education have a higher rate of ANC visits compared to women with no education. This study is consistent with nationally representative data collected in 2020 in Ethiopia (24), a systematic review, and meta-analysis conducted in Ethiopia (25) and in East Africa (14, 26). Also, the Demographic and Health Survey (DHS) data from Mauritania (8), Cameron (27), Gahan (28), and Sub-Saharan Africa (29) are in agreement with the finding of this study. With more education, women would become more informed when making healthcare decisions and would be more conscious of the usage of ANC services and their effects. Also, as women's educational levels increase, their knowledge of obstetric complications increases, which encourages them to use ANC services (30). Education provides the skill and knowledge required for accessing information and initiates women to receive health-related messages; on top of that, it facilitates communication with health care providers (31).

The number of ANC visits increases with maternal age. In comparison to women in the 15–19 year age group, more ANC visits were made by women in the 20–24 year, 25–29 year, 30–34 year, 35–39 year, and 40–44 year age groups. Nationally representative data collected in Ethiopia in 2020 support this (24), a systematic review and meta-analysis carried out in Cameroon (27), the DHS of Sub-Saharan Africa (29), and East African countries (26). This might be a result of the fact that women are more likely to use healthcare services as their experience and knowledge of them grows (32, 33).

Religion is significantly associated with the number of ANC visits. Women who were protestant, Muslim, traditional, or practiced another religion had fewer ANC visits than Orthodox believers. This is supported by findings from Nigeria (34) and Ghana (35). This needs additional evidence and a more qualitative approach to explore the effect of religion on the utilization of ANC.

ANC visits were higher for households in the middle or rich household wealth index than for those in the lower wealth index. This study is similar to systematic reviews and meta-analyses conducted in East African countries (26), the DHS of Mauritania (8), Cameron (27), Gahan (28). and Sub-Saharan Africa (29). Health-seeking behavior depends largely on economic factors. Income is important for purchasing health care. This affects women's ability to seek ANC services (36). Lack of income limits ANC visits, and most of them visit the service late in pregnancy (37). Even though in Ethiopia antenatal services are offered without charge, indirect costs like transportation costs to and from health facilities limit service utilization among women with low wealth index. Women in high wealth indexes also get the service from private health institutions with direct payments (38).

Women who delivered their last birth in a public health institution, a private health institution, or an NGO health facility had a higher number of ANC visits compared to those who delivered at home. This is supported by a study from Bangladesh (39) and a systematic review conducted in Sub-Saharan Africa (37).

Women are less likely to seek ANC services as household sizes increase. Compared to households with less than five members, women who live in households with more than ten people experienced fewer ANC visits. This study is in line with studies from Rwanda (40) and North West Ethiopia (41). This could be because as families become larger, financial difficulties arise, preventing the women from accessing ANC. Also, when families get larger, women take on more household duties and spend more time caring for their children, which have an impact on health care seeking behavior (42).

Since we have used the national data, which is collected from both urban and rural areas in nine regions and two city administrations in Ethiopia, the study was thought to be more representative than other studies. But the study might have faced the following limitations. There might be recall bias since the data was collected from women based on their last birth history. Due to the nature of the data, we might have missed important variables such as those related to the husband, previous exposure variables, and paternal variables.

## Conclusion

In Ethiopia, there is the highest regional disparity in the number of ANC visits. The number of ANC visits was influenced by the mother's age, education, religion, household wealth index, place of delivery, and household size. The government of Ethiopia should focus in increasing an access to health care service in areas with the fewest ANC visits in order to reduce inequities between regions in Ethiopia. Regarding the ANC visits, there should be initiatives that address the demands of pastoralist and agro-pastoralist communities to increase ANC utilization. As with many other health outcomes, education and low socio-economic status were associated with low ANC visit but these are tied to the overall social development of a country and are not immediately amenable to public health interventions.

## Data Availability

The original contributions presented in the study are included in the article/Supplementary Material, further inquiries can be directed to the corresponding author.
